# Ginsenoside compound K promotes β-amyloid peptide clearance in primary astrocytes via autophagy enhancement

**DOI:** 10.3892/etm.2014.1885

**Published:** 2014-08-06

**Authors:** JINHUI GUO, LI CHANG, XIN ZHANG, SUJUAN PEI, MEISHUANG YU, JIANLIAN GAO

**Affiliations:** 1 Department of Pharmaceutics, The First Affiliated Hospital of Xinxiang Medical University, Weihui, Henan 453100, P.R. China; 2 Department of Neurology, The First Affiliated Hospital of Xinxiang Medical University, Weihui, Henan 453100, P.R. China

**Keywords:** Alzheimer’s disease, compound K, β-amyloid peptides, autophagy

## Abstract

The aim of the present study was to investigate the effect of ginsenoside compound K on β-amyloid (Aβ) peptide clearance in primary astrocytes. Aβ degradation in primary astrocytes was determined using an intracellular Aβ clearance assay. Aggregated LC3 in astrocyte cells, which is a marker for the level of autophagy, was detected using laser scanning confocal microscope. The effect of compound K on the mammalian target of rapamycin (mTOR)/autophagy pathway was determined using western blot analysis, and an enzyme-linked immunosorbent assay was used for Aβ detection. The results demonstrated that compound K promoted the clearance of Aβ and enhanced autophagy in primary astrocytes. In addition, it was found that phosphorylation of mTOR was inhibited by compound K, which may have contributed to the enhanced autophagy. In conclusion, compound K promotes Aβ clearance by enhancing autophagy via the mTOR signaling pathway in primary astrocytes.

## Introduction

Alzheimer’s disease (AD) is a progressive neurodegenerative disease of the central nervous system, with memory loss as the primary clinical manifestation. As a result of the aging population, the number of patients with AD is predicted to be >100 million in 2050 worldwide (1). However, at present, there are no effective drugs that delay the progression of AD.

Previous studies have demonstrated that β-amyloid (Aβ) peptides have an important role in AD. Aggregation of Aβ may cause neurofibrillary tangles, inflammation and neuronal loss, resulting in the development of AD (2). It is thought that reduced generation or accelerated clearance of Aβ may delay the pathological progression of AD (3,4). Autophagy is considered to be an important regulatory factor in Aβ clearance. During autophagy, Aβ is taken up by autophagic vacuoles, which then fuse to the lysosome and are degraded (5,6). Therefore, autophagy enhances Aβ clearance. It has been previously demonstrated that mammalian target of rapamycin (mTOR) kinase is an important energy sensitivity factor. mTOR is inhibited when there is lack of energy, and inhibition of mTOR results in the activation of autophagy to produce additional energy (7,8). Therefore, inhibition of mTOR may enhance autophagy, and as a result increase the clearance of intracellular Aβ, thereby delaying the pathological progression of AD (9).

*Panax ginseng* is a traditional Chinese medicine, which has been used as a medicine for thousands of years. Studies have shown that ginseng has a number of biological activities, including as an antioxidant, anti-aging agent, inhibitor of cell apoptosis and cognition enhancer (10,11). Ginsenosides are active compounds extracted from ginseng, and it has been demonstrated that ginsenoside Rg1 is able to improve memory and has anti-dementia effects (12). Ginsenoside compound K is a metabolite of panaxadiol (a saponin) that is generated by the metabolic action of intestinal flora in humans. It is considered that numerous ginsenosides are metabolized into compound K prior to becoming active *in vivo*. Compound K is hypothesized to be the active compound *in vivo*, while ginsenosides may be prodrugs that have no effect (13). Therefore, the pharmacological mechanisms of compound K require further investigation. In present study, the Aβ-scavenging effect of compound K and the underlying mechanisms were investigated in primary astrocytes.

## Materials and methods

### Primary culturing of mouse astrocytes

C57 mice were purchased from the Experimental Animal Center of Xinxiang Medical University (Xinxiang, China) and at 18 days of pregnancy were anaesthetized using pentobarbital and then paunched. The fetal mice were removed and placed into pre-cooling D-Hank’s buffer. The brains of the fetal mice were removed and the meninges were discarded. The cerebral cortex was separated from the brain tissues and then digested for 15 min at 37°C in 8 ml 0.125% trypsin (2.5% trypsin diluted 20-fold with D-Hank’s and DNase, at a final concentration of 0.2 mg/ml). Digestion was terminated by the addition of 8 ml Dulbecco’s modified Eagle’s medium (Gibco, Grand Island, NY, USA) containing 10% horse serum (Gibco) for 5 min. The upper suspension was transferred to a 50-ml tube and an additional 8 ml complete growth medium was added to the deposit and the previous step was repeated. The cells in the suspension were placed at a density of 2×10^5^ cell/cm^2^ in a 75-cm^2^ flask that was pre-coated with poly-D-lysine. The cells were incubated at 37°C and 5% CO_2_ for 9 days, and the medium was replaced every three days. The flask was then placed on a ZHWY-110X shaker table (Zhicheng Co., Shanghai, China) at 200 rpm at 37°C overnight. On the second day, the flask was rinsed twice with D-Hank’s at 37°C, and the remaining adherent cells were collected with trypsin and subcultured. Following attachment, the cells were treated with Ara-C for 96 h (medium renewal once) at a final concentration of 10 μM, and the cells obtained were astrocytes. This study was performed in strict accordance with the recommendations in the Guide for the Care and Use of Laboratory Animals of the National Institutes of Health (8th edition, Bethesda, MD, USA, 2010). The animal use protocol was reviewed and approved by the Institutional Animal Care and Use Committee of the First Affiliated Hospital of Xinxiang Medical University (Weihui,, China).

### Detection of aggregated LC3

A viral expression vector containing a red fluorescent protein-LC3 construct (Jikai Biotechnology Co. Ltd., Shanghai, China) was transfected into the primary astrocytes. Transfection was performed using a Sunma-sofast gene transfection kit according to the manufacturer’s instructions (Sunma Biotechnology Co., Ltd., Xiamen, China). LC3 proteins were then confirmed to be stably expressed in the cells. After two days, the cells were treated with 50 μM compound K (Fangcheng Biotechnology Co. Ltd., Beijing, China) for 2 h, and the aggregation state of the LC3 proteins was detected by confocal laser scanning microscopy (TCS-SP5, Leica, Bensheim, Germany).

### Western blot analysis

Cells treated with different concentration of compound K were treated with radio-immunoprecipitation assay lysis buffer at 75°C for 10 min. The lysates were incubated at 99°C for 15 min, and then centrifuged at 10,000 × g for 5 min at 4°C. The supernatant was separated using SDS-PAGE and then transferred to a polyvinylidene difluoride membrane under semi-dry electrotransfer conditions. The membrane was incubated in blocking buffer [1X Tris-buffered saline (TBS), 0.5% Tween 20 and 5% skimmed milk powder] for 30 min, followed by incubation with the primary antibody (targeting P70S6K, P70S6K-P, ULK1, ULK1-P, mTOR, mTOR-P, P62 or GAPDH; Cell Signaling Technology Inc., Beverly, MA, USA) at 4°C overnight. The membranes were then rinsed 3 times with TBS and Tween 20 (TBST) for 15 min, and then incubated with the corresponding monoclonal, anti-rabbit secondary antibody (Santa-Cruz Biotechnology Inc., Santa Cruz, CA, USA) for 2 h, prior to being rinsed again with TBST 3 times. The membrane was then incubated with SuperSignal West Dura Chemiluminescent Substrate (Pierce, Thermo Scientific, Rockford, IL, USA) for 5 min and images were captured by X-ray film exposure.

### Aβ clearance assay

The Aβ clearance assay in primary astrocytes was performed as previously described by Cramer *et al* (14). Briefly, the cells were incubated with different concentrations of compound K (0, 1, 10, 20 and 50 μM) for 18 h, meanwhile, chloroquine (an inhibitor of autophagy) was used as a control and then exogenous Aβ (Invitrogen Life Technologies, Carlsbad, CA, USA) was added to a final concentration of 2 μg/ml. The cells were then incubated for a further 3 h. The cells were washed 3 times with phosphate-buffered saline, and then treated with lysis buffer (50 mM Tris and 1% SDS) at 37°C for 15 min. The lysates were centrifuged at 12,000 × g for 15 min, and the supernatant was collected. Aβ was then quantified using an enzyme-linked immunosorbent assay (ELISA kit for Aβ detection).

### ELISA

An ELISA for Aβ detection was conducted in accordance with the manufacturer’s instructions (Invitrogen Life Technologies). The diluted samples were incubated with Aβ antibody in a 96-well plate that was pre-coated with Aβ antibody. After 3 h, the plate was rinsed with cleaning solution (Biyuntian Co., Shanghai, China) four times, and then incubated with the secondary antibody for 30 min and rinsed five times. The chromogenic substrate was then added and the plates were incubated for a further 30 min. Finally the reaction was terminated using stop solution. The intensity of color developed was measured using microplate reader (Bio-Rad 680, Bio-Rad, Hercules, CA, USA) at 570 nm. In order to eliminate the interference of the cell density, the cells were lysed (50 mm Tris-HCl, 0.15 M sodium chloride, 1% P40 and 0.1% SDS) and the protein content was measured using the bicinchoninic acid assay method. The measured density was adjusted according to the total protein content.

### Statistical analysis

The data are expressed as the mean ± standard deviation and were analyzed using SPSS software, version 16.0 (SPSS, Inc., Chicago, IL, USA). One-way analysis of variance was used to compare the scores of different groups. P<0.05 was considered to indicate a statistically significant difference.

## Results

### Compound K promotes clearance of Aβ in primary astrocytes

**Figure 1:**
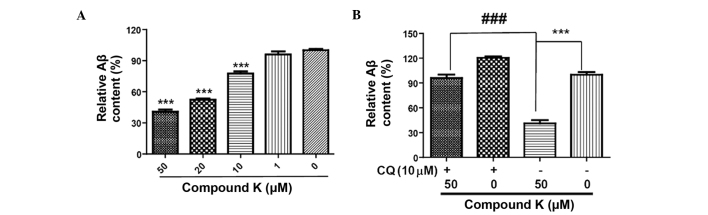
Compound K promotes clearance of Aβ in primary astrocytes. (A) Primary astrocytes were treated with different concentrations of compound K (50, 20, 10, 1 and 0 μM) for 18 h. Exogenous Aβ was then added and the cells were incubated for a further 3 h. Then the cells were lysed and the content of Aβ was measured by ELISA. (B) Primary astrocytes were treated with 50 μM compound K or 10 μM chloroquine or a combination of the two for 18 h. Exogenous Aβ was added and the cells were incubated for 3 h. The cells were then lysed and the Aβ content was measured by ELISA (n=3). ^***^P<0.001 compared with the 0 μM compound K group, ^###^P<0.001.

The levels of Aβ in astrocytes treated with compound K were significantly lower compared with those in untreated astrocytes. The differences were significantly different for the 10, 20 and 50 μM concentrations of compound K (P<0.001; Fig. 1). These results indicate that compound K enhances Aβ clearance in primary astrocytes. In order to investigate the association between compound K and autophagy, chloroquine, an inhibitor of autophagy, was used as a control. The results demonstrated that chloroquine markedly attenuated the effect of compound K on the enhancement of Aβ clearance. This indicates that compound K promotes Aβ clearance through the enhancement of autophagy in primary astrocytes.

### Compound K enhances autophagy in primary astrocytes

**Figure 2:**
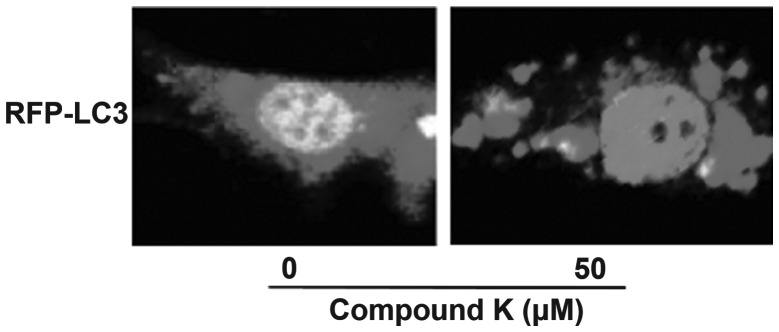
Compound K enhances autophagy in primary astrocytes. Primary astrocytes were transfected with LC3 and and stably expressed the protein. After 2 days, cells were incubated with 50 μM compound K for 2 h. The aggregation state of LC3 was then detected by laser scanning confocal microscopy.

Autophagy has an important role in Aβ clearance. In order to further clarify the association between autophagy and Aβ clearance, the effect of compound K on autophagy was investigated. LC3 is an important marker of autophagosome formation. When autophagy is enhanced, LC3 proteins that are dispersed throughout the cell gather to the membrane of autophagosome, which can then be detected using laser scanning confocal microscopy. Following transfection with LC3, the primary astrocytes were found to stably express LC3 protein. It was observed that LC3 proteins aggregated following incubation with 50 μM compound K for 2 h. This indicates that compound K enhances autophagy in astrocytes, which may contribute to increased clearance of Aβ (Fig. 2).

### Compound K enhances autophagy by inhibiting the mTOR signal pathway

**Figure 3:**
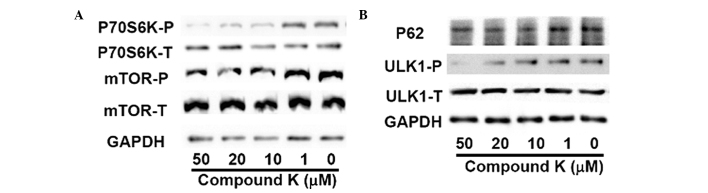
Compound K enhances autophagy by inhibiting the activity of mTOR. (A) Primary astrocytes were treated with different concentrations of compound K (50, 20, 10, 1 or 0 μM) for 24 h. The total proteins were collected and the phosphorylation levels of P70S6K and mTOR were detected by western blot analysis. (B) Primary astrocytes were treated with different concentrations of compound K (50, 20, 10, 1 or 0 μM) for 24 h. The total proteins were collected and the phosphorylation of ULK1 and the levels of P62 were detected using western blot analysis. mTOR, mammalian target of rapamycin.

As an important factor of energy sensitivity, mTOR kinase is inhibited when there is an energy shortage, leading to an enhancement of autophagy. Therefore, the phosphorylation levels of mTOR and P70S6K, the substrate of mTOR, in primary astrocytes treated with compound K were investigated by western blot analysis. As shown in Fig. 3A, the phosphorylation levels of mTOR and P70S6K were reduced by compound K, indicating that compound K enhances autophagy by inhibiting the activity of mTOR. To further investigate this, the phosphorylation of ULK1, the major initiator protein of autophagy, and the levels of P62, the marker of the autophagy, were then investigated (15). As shown in Fig. 3B, compound K significantly reduced the expression level of P62 and the phosphorylation of ULK1, indicating that compound K activates and enhances autophagy in astrocytes.

These results suggest that compound K may have an inhibitory effect on the phosphorylation of mTOR and subsequently enhance autophagy, which may contribute to increased scavenging of Aβ in primary astrocytes.

## Discussion

Aβ is considered to be one of the key proteins in AD progression. *In vivo,* Aβ is in a dynamic equilibrium between generation and scavenging, and excess generation or weak scavenging of Aβ is the main pathogeny of AD. Drugs that reduce Aβ production or increase the clearance of Aβ may slow AD progression. Autophagy is the most important pathway for the clearance of abnormal molecules, cell subunits and abnormal aggregates of proteins. Autophagy levels have been found to be significantly decreased in various neurodegenerative diseases, including AD and Parkinson’s disease (16,17). Therefore, enhanced autophagy may increase the clearance of Aβ, which may delay the pathological process of AD. In a previous study it was reported that resveratrol and its derivatives from grape seeds enhance autophagy through AMPK activation and subsequently the inhibition of mTOR, resulting in the scavenging of Aβ, which may have a role in the treatment of AD (18). In addition, latrepirdine, a type of antihistamine that is already on the market, has been demonstrated to increase the clearance of Aβ by enhancing autophagy, and has a role in the treatment of AD (19). Therefore, compounds that are able to enhance the scavenging of Aβ by promoting autophagy may be effective in AD therapy, and the screening of this type of compound is valuable for the treatment of AD.

Previous studies have shown that *Panax ginseng* has a number of biological activities, including the ability to improve memory. Compound K is the metabolite of numerous ginsenosides in the intestine, and is the main active entity of *Panax ginseng in vivo*. It has previously been shown that ginsenosides are able to upregulate the expression of brain-derived neurotrophic factor and inhibit the phosphorylation of Tau, which results in slowing of the formation of neurofibrillary tangles and should improve learning ability and memory. In addition, it has been shown to have an anti-AD role in a rat model of AD (20).

The effect of compound K on Aβ scavenging has yet to be fully elucidated. In the present study, the effect of compound K on Aβ scavenging and the mechanisms involved was investigated using the Aβ clearance assay *in vitro*. The results demonstrated that compound K significantly enhanced the clearance of Aβ in primary astrocytes. Furthermore, it was shown that the phosphorylation of mTOR was inhibited by compound K, which may contribute to enhanced autophagy, and thereby increase the scavenging of Aβ. In conclusion, these results indicate that compound K may increase the clearance of Aβ from astrocytes and then slow the pathological progression of AD. This study provides an important basis for the use of *Panax ginseng* as a traditional Chinese medicine for improving memory, and provides a novel interpretation of the mechanism of compound K in AD therapy.
